# Adipocyte‐specific Krüppel‐like factor 14 overexpression confers sex‐biased protection from weight gain on a high‐fat diet

**DOI:** 10.14814/phy2.70513

**Published:** 2025-08-11

**Authors:** Yonathan Tamrat Aberra, Qianyi Yang, Ashlyn Cowan, Bilge Yaylak Gediksiz, Mitchell Thomas, David Gragirenes‐Delgado, Foday Keita, Mete Civelek

**Affiliations:** ^1^ Department of Biomedical Engineering University of Virginia Charlottesville Virginia USA; ^2^ Department of Biology University of Puerto Rico–Cayey Cayey Puerto Rico USA; ^3^ Department of Biology Howard University Washington District of Columbia USA

**Keywords:** adipose tissue, insulin resistance, metabolic syndrome, obesity, sex differences

## Abstract

Metabolic syndrome, a constellation of cardiometabolic risk factors including abdominal obesity, predisposes individuals to type 2 diabetes and cardiovascular disease. One frequently replicated MetSyn genetic association signal is located near KLF14 and is linked to central adiposity, with stronger effects observed in females. Lower KLF14 expression has been associated with detrimental metabolic phenotypes; however, the therapeutic effect of KLF14 germline overexpression remains unexplored. Here, we generated an adipocyte‐specific Klf14 overexpression mouse model to investigate its role in metabolic regulation. Transgenic overexpression conferred sex‐dependent metabolic benefits, including reduced weight gain, improved body composition, and enhanced acute insulin sensitivity, predominantly in female mice. These effects were accompanied by increased expression of genes involved in lipid uptake and thermogenic browning of white adipose tissue. Our findings demonstrate that KLF14 overexpression confers protective metabolic effects in a sex‐specific manner and support the potential of KLF14‐targeted strategies for treating metabolic syndrome.

## 1 INTRODUCTION

Metabolic syndrome (MetSyn) is a major risk factor for cardiovascular disease and type 2 diabetes, and its prevalence is increasing (Lusis et al., 2008). The diagnostic criteria for MetSyn require three of the five following conditions: abdominal obesity, hypertension, high blood glucose, high serum triglycerides, and low serum high‐density lipoprotein (HDL) (Grundy et al., 2005). The conditions associated with MetSyn are complex traits with genetic heritability (van Dongen et al., 2013), enabling genome‐wide association studies (GWAS). GWAS have identified hundreds of genetic loci associated with MetSyn traits (Aragam et al., 2022; Chen et al., 2021; Graham et al., 2021; Pulit et al., 2019; Suzuki et al., 2024; Tcheandjieu et al., 2022). Most genetic loci associated with these traits are located in noncoding regions of the genome, where their effector mechanisms are likely through the genetic regulation of gene expression. Genetic variants that alter the expression of proximal (*cis*) genes and distal (*trans*) genes are called quantitative trait loci (QTL). The integration of GWAS with QTL studies with colocalization analysis can provide specific hypotheses about the genetic regulatory mechanisms at GWAS loci (Aberra et al., 2023; Giambartolomei et al., 2014; Plagnol et al., 2009; Wallace, 2020, 2021; Wallace et al., 2012).

The MetSyn genetic association signal at 7q32.3 lies 45 kb upstream of the gene encoding transcription factor Krüppel‐like factor 14 (KLF14) (Small et al., 2018). The risk variant in the 7q32.3 locus is associated with lower expression of KLF14 specifically in adipose tissue. The risk variant is highly pleiotropic; it is associated with abdominal obesity, serum triglyceride levels, type 2 diabetes, and coronary artery disease (Aragam et al., 2022; Graham et al., 2021; Small et al., 2018; Suzuki et al., 2024; Tcheandjieu et al., 2022). These associations are sex‐biased, with more pronounced effects and associations in women than in men (Yang & Civelek, 2020). The KLF14 *cis*‐eQTL is associated with *trans* effects on nearly 400 genes genome‐wide. Genes in the KLF14 *trans*‐eQTL network are enriched for metabolic pathways, cholesterol biosynthesis, and lipid metabolism, suggesting the role of KLF14 as a master regulator of adipose tissue biology. Follow‐up studies in humans and rodents have shown that KLF14 expression is linked to various adipocyte biology phenotypes. In women, homozygous risk variant carriers have larger adipocytes, and knockdown of KLF14 inhibited preadipocyte maturation into adipocytes (Small et al., 2018). In female mice but not male mice, adipocyte‐specific KLF14 depletion resulted in insulin resistance, increased adiposity, and fat redistribution from subcutaneous to visceral adipose tissues (Yang et al., 2022), characteristic of abdominal obesity.

Taken together, previous studies pointed to the sex‐biased deleterious effects of KLF14 depletion. However, it remains unclear if germline KLF14 overexpression is protective. Yang et al. developed a transgenic mouse model of adipocyte‐specific KLF14 overexpression (KLF14Tg) and found that KLF14Tg mice were protected from adiposity on a high‐fat diet in female KLF14Tg mice (Yang et al., 2022). We further investigated the effect of KLF14 overexpression on adiposity, insulin resistance, serum lipid profiles, and gene expression in KLF14Tg mice. We found broad as well as sex‐biased protection from weight gain, adiposity, and insulin resistance in our model of KLF14 overexpression. Accompanying these changes, we found that genes governing lipid uptake and adipose tissue browning were expressed at greater levels by KLF14Tg mice. Our findings point to the therapeutic potential of adipose KLF14 overexpression and may pave the way for novel MetSyn therapeutics.

## 2 MATERIALS AND METHODS

### 2.1 Generation of Klf14Tg mouse line

As previously described (Yang et al., 2022), to generate adipocyte‐specific Klf14‐overexpressing mice (Klf14Tg), Klf14 cDNA was PCR‐amplified from C57BL/6J and cloned downstream of the 5.43‐kb Adipoq promoter into the pADNpcDNA3.1 KanR vector using Gibson Assembly. The 8.2‐kb expression cassette was excised with NotI and NgoMIV, gel‐purified, and injected into the pronuclei of fertilized C57BL/6N eggs at the University of California, Irvine Transgenic Mouse Facility.

### 2.2 Animal husbandry and care

All animal protocols were approved by the University of Virginia Animal Care and Use Committee. Mice were maintained in a 12‐h light/12‐h dark cycle with free access to water and standard chow after weaning and before week 12 (Envigo Teklad LM485 irradiated mouse/rat sterilizable diet, Cat No. 7912). Starting at 12 weeks of age, mice were fed a rodent diet with 40% kcal fat, 43% kcal carbohydrate, and 17% kcal protein (Research Diets, Catalog Number D12079B). We determined the genotypes of mice with the KAPA Mouse Genotyping Kit (Millipore Sigma KR0385_S). We isolated DNA from tail clippings and used PCR with primers targeting the adiponectin promoter region and gene body of KLF14 (Table S1). PCR‐amplified DNA was then separated with gel electrophoresis, and images were taken. Transgenic overexpression mice were identified by the presence of two amplified signals for KLF14, the endogenous KLF14 gene, and the transgenic overexpression cassette. We included 55 mice of approximately equal proportions: male, female, transgenic, and wild‐type (Table S2). At the end of the experiments, mice were euthanized by cervical dislocation after induction of deep anesthesia using isoflurane, consistent with the recommendations of the Panel of the American Veterinary Medical Association.

### 2.3 Body weight and composition analysis

The mice were weighed; then the total body fat and lean mass were assessed with a noninvasive quantitative MRI system (Echo Medical Systems, Catalog Number EchoMRI 3‐in‐1 v2.1) every 4 weeks. Differences between groups were identified with the repeated measures ANOVA, as described previously in the literature (Nixon et al., 2010).

### 2.4 Insulin and glucose tolerance tests

Glucose and insulin tolerance tests were performed as previously described (Fantin et al., 2000). For the glucose tolerance test, mice were fasted from 7:30 a.m. to 1:30 p.m. and were administered glucose at 1 mg/g body weight intraperitoneally. Blood glucose levels were measured with a OneTouch Ultra Glucometer (LifeScan) in blood drops obtained from nicked tail veins before and at 20, 40, 60, 90, and 120 min after the injection. The insulin tolerance test was performed at 1:30 p.m. on fasted mice. Insulin (100 units/mL; Humulin, Eli Lilly) at 0.75 units/kg body weight was administered by intraperitoneal injection of 0.25 units/mL solution in 0.9% NaCl. Blood glucose levels were measured with a OneTouch Ultra Glucometer (LifeScan) in blood drops obtained from nicked tail veins before and at 20, 40, 60, 90, and 120 min after the injection. Hypoglycemic mice were excluded from the study and received care. Differences between groups were identified with the repeated measures ANOVA.

### 2.5 Primary isolation of tissues and cells

Transcardiac perfusion was performed immediately after sacrifice. We isolated and weighed the heart, kidneys, liver, skeletal muscle from the hind legs, subcutaneous white adipose tissue (sWAT), and perigonadal WAT (pWAT) from each mouse. Mature adipocytes and the stromal‐vascular fraction from each fat pad were isolated as previously described (Kumar et al., 2010). Briefly, fat pads were placed in Krebs Ringer HEPES (KRH)–BSA buffer containing collagenase type I (1 mg/mL, 2 mg/g of tissue; Worthington Biochemical Corp.) and minced with scissors. Small tissue pieces were incubated in a 37°C shaking water bath (100 rpm) for 1 h. Fat cells were separated from nonfat cells and undigested debris by filtration through a 150 μm CellTrics filter (Fisher Scientific Catalog Number NC9021438) and four washes by flotation with KRH buffer without BSA. After centrifugation at 1000 g for 15 min at 4°C, the isolated fat cells floating on the surface were used for further analysis. Tissues and cells isolated from mice were split into equal parts for total RNA extraction and preparation of lysates for immunoblotting.

### 2.6 RNA extraction, PCR, and quantification

Total RNA was isolated from adipocytes using a combination of TRIzol Reagent (Thermo Fisher Scientific, Waltham, MA) and RNeasy kit (Qiagen, Hilden, Germany) according to the manufacturer's instructions (RNeasy® Mini Handbook, 2023). Adipocytes isolated from adipose tissue were homogenized in 2 mL of TRIzol Reagent using the Tissue‐Tearor homogenizer (Model 985370, BioSpec Products). After homogenization, samples were incubated at room temperature for 5 min and centrifuged at 12,000 g for 10 min at 4°C. The resulting fat monolayer on top was carefully avoided when pipetting the rest of the sample into a clean tube. Then, 400 mL of chloroform was added, and samples were mixed by vortexing. Samples were kept at room temperature for 3 min before centrifugation at 12,000 g for 30 min at 4°C. After centrifugation, samples were separated into three phases, with the RNA in the upper phase. The RNA phase was transferred to a new tube without disturbing the interphase. Sample volumes were measured, and 1.5× sample volumes of 100% ethanol were added. Samples were mixed thoroughly by inverting the tubes several times. Samples were then loaded on miRNeasy spin columns (Qiagen), and the manufacturer's protocol was followed for subsequent steps. Total RNA concentrations were quantified using a Qubit Fluorometer and a Qubit RNA BR assay kit (Thermo Fisher Scientific). Then, 1 mg of total RNA was reverse transcribed using SuperScript IV reverse transcriptase (Invitrogen). Real‐time PCR was performed in the QuantStudio 5 Real‐Time PCR System (Thermo Fisher Scientific) using SYBR Green Master Mix (Roche) and gene‐specific primers (Table S3) or with the TaqMan gene‐specific assays for Gapdh and Klf14 (Thermo Fisher Scientific Catalog Numbers 4331182 and 4351372).

### 2.7 Serum lipid quantification

Mice were fasted for 6 hours from 7:30 a.m. to 1:30 p.m. before serum samples were taken. Blood samples were collected through retro‐orbital bleeding. For HDL, total cholesterol, and TG measurements, plasma was obtained using heparinized capillary tubes, followed by centrifugation at 7800 relative centrifugal force in a microfuge for 10 min at 4°C. Total cholesterol (TC), HDL, and TG levels were measured using colorimetric assays (FUJIFILM Wako Diagnostics, Cat Nos. 999‐02601, 997‐72591, and 464‐01601). We calculated LDL from TC, HDL, and TG levels using the Friedewald formula (Friedewald et al., 1972):

$$\mathrm{LDL}-C=\left(\mathrm{TC}\right)-\left(\mathrm{HDL}-C\right)-\left(\mathrm{TG}/5\right).$$

### 2.8 Indirect calorimetry analysis

Mice were placed in Oxymax metabolic chambers (Comprehensive Laboratory Animal Monitoring System [CLAMS] from Columbus Instruments), under constant environmental conditions: temperature (22°C), 12‐h light and 12‐h dark cycle, and constant airflow, as previously described (Wortley et al., 2005). VO_2_, VCO_2_, and ambulatory activity were measured every 5 min in each mouse for 72 h. Mice were acclimated for 24 h, and measurements taken in the next 48 h were used for data analysis. (1) Respiratory Exchange Ratios (RERs), (2) Carbohydrate Utilization (CHU), and (3) Fat Utilization (FU) were calculated as follows (Fentz et al. 2015):

1. RER = VCO_2_/VO_2_,
2. CHU = 20kJ/L * VO_2_ * (RER−0.7)/0.3, and
3. FU = 20kJ/L * VO_2_ * (RER−0.3)/0.7.

Mice in each chamber had free access to water and food. Locomotor activity was monitored by a multidimensional infrared light beam system surrounding each cage; quantified by ambulatory beam breaks in the X and Y dimensions.

## 3 RESULTS

### 3.1 KLF14Tg mice are broadly protected against diet‐induced adiposity and weight gain

**FIGURE 1:**
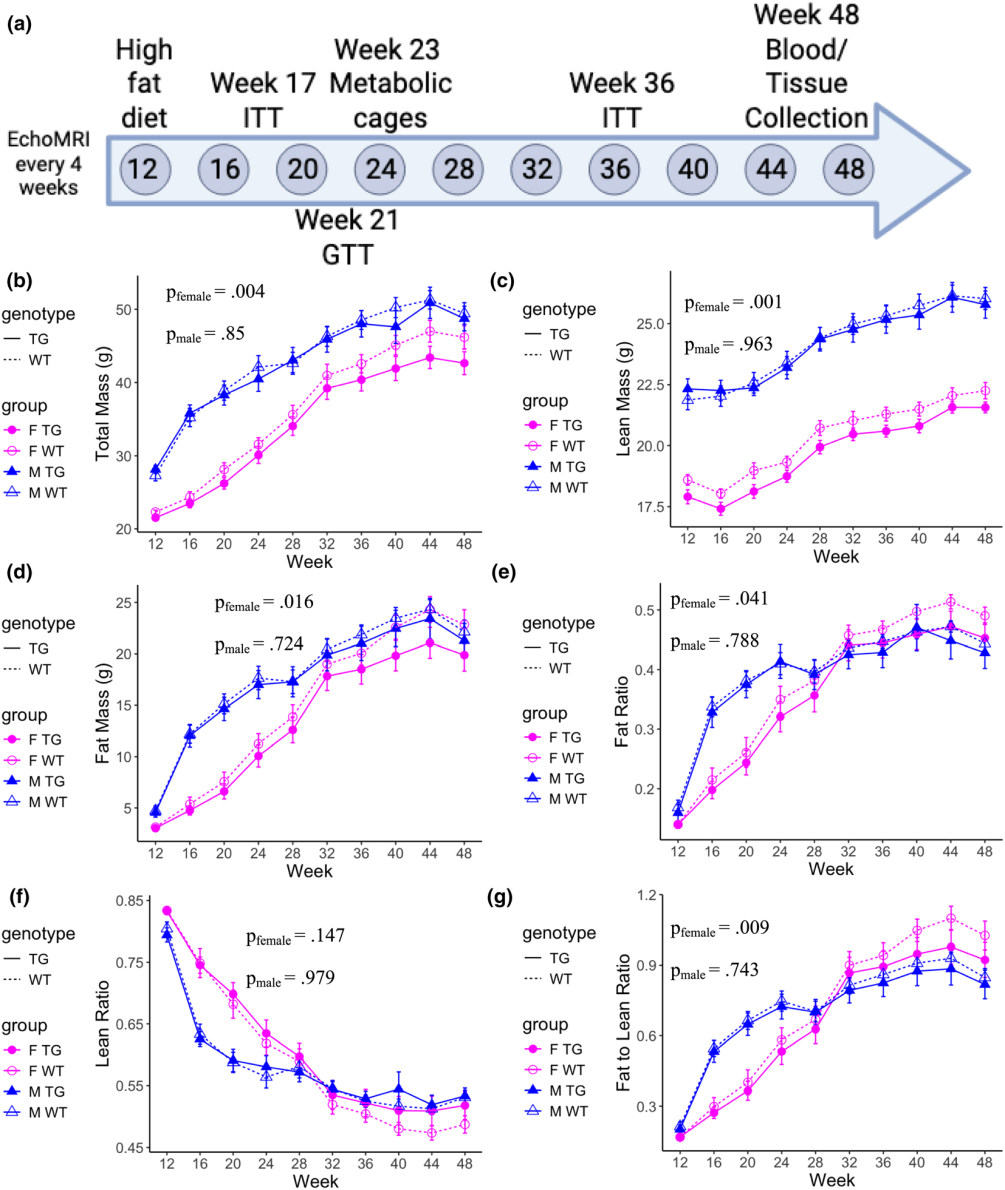
Transgenic mice are protected from diet‐induced weight gain. (a) Mouse characterization pipeline. Female Klf14Tg mice (F TG) (*n* = 16), female wild‐type mice (F WT) (*n* = 12), male Klf14Tg mice (M TG) (*n* = 11), and male wild‐type mice (M WT) (*n* = 16) were put on a high‐fat diet (MHF Diet, 32.5 kcal% Fat) from week 12 to the conclusion of the study at week 48. (b) Mean total mass of mice in each group. (c) Mean lean mass of mice in each group. (d) Mean fat mass of mice in each group. (e) The mean ratio of fat to total mass in each group. (f) The mean ratio of lean to total mass in each group. (g) The mean ratio of fat to lean mass in each group. Each is presented with the standard error of the mean (SEM). Differences were assessed with the repeated measures ANOVA with terms for sex, genotype, and the interaction between sex and genotype. The presented *p* value is for the difference in genotype.

Mice were weaned at 3 weeks of age and maintained on standard chow until 12 weeks, after which they were transitioned to a high‐fat diet (HFD). Body weight and composition were assessed every 4 weeks through 48 weeks of age (Figure 1a). KLF14Tg mice exhibited significantly reduced weight gain compared to wild‐type (WT) littermates, with more pronounced effects in females (average reduction of 2.07 g across all time points) than in males (0.51 g) (Figure 1b). By 48 weeks, female KLF14Tg mice weighed 3.50 g less than WT controls, whereas the difference in males was 0.67 g. This reduction in body mass was primarily driven by differences in fat mass accumulation.

Female KLF14Tg mice had, on average, 0.66 g less lean mass than WT females across all time points; this difference was not observed in males (Figure 1c). Fat mass differences became more pronounced over time, with female KLF14Tg mice accumulating 1.58 g less fat on average than WT controls, compared to a 0.56 g difference in males (Figure 1d). By 48 weeks, the reduction in fat mass reached 2.96 g in females and 0.85 g in males. The ratio of fat mass to total mass was lower in female transgenic mice compared to female wild‐type mice, but this difference was not observed in male mice (Figure 1e). Neither male nor female mice showed differences in their ratios of lean mass to total mass (Figure 1f). The adiposity of female transgenic mice was lower than that of their wild‐type littermates only (Figure 1g). Taken together, these data suggest that the attenuation of weight gain in KLF14Tg mice is primarily attributable to reduced adiposity, particularly in females.

Tissue mass measurements—including visceral and subcutaneous fat, liver, kidney, heart, tibia, and skeletal muscle—revealed no significant differences between genotypes, even after normalization to tibia length (Figure S1). Together, these findings indicate that adipocyte‐specific Klf14 overexpression confers protection against diet‐induced adiposity in female mice, but not in male mice.

### 3.2 KLF14Tg mice are transiently protected from glucose and insulin resistance

**FIGURE 2:**
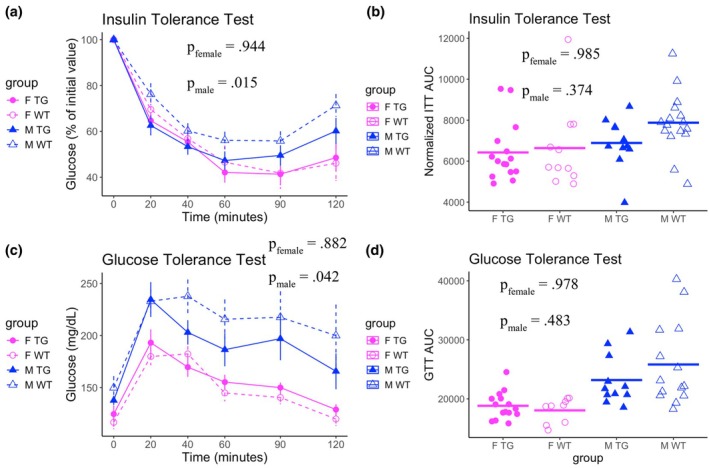
Transgenic mice are transiently protected from diet‐induced glucose and insulin resistance. (a) The mean of blood glucose levels, normalized to the initial time point, is plotted, along with the standard error of the mean (SEM). (b) The area under the curve (AUC) for the time‐course blood glucose levels in the insulin tolerance test. (c) The mean of blood glucose levels, normalized to the initial time point, is plotted, along with the SEM. (d) The AUC for the time‐course blood glucose levels in the glucose tolerance test. Glucose and insulin tolerance tests were performed on F TG (*n* = 16), F WT (*n* = 12), M TG (*n* = 11), and M WT (*n* = 16) mice. Differences in time‐course glucose levels were assessed with the repeated measures ANOVA with terms for sex, genotype, time, and interactions between these. This was followed by Tukey's test comparing wild‐type and transgenic mice within sex. Differences in the AUC glucose levels were assessed with the repeated measures ANOVA, followed by Tukey's test comparing wild‐type and transgenic mice within sex.

To assess systemic insulin sensitivity, we performed intraperitoneal insulin tolerance tests (ITTs) at 17 and 36 weeks of age. Mice were administered insulin at doses normalized to body weight, and blood glucose was measured at baseline and 20, 40, 60, 90, and 120 min post‐injection. KLF14Tg mice were more insulin sensitive than wild‐type (WT) controls at 36 weeks, with a more pronounced effect in males (Figure 2a). This genotype‐dependent difference was not observed at 17 weeks (Figure S2), suggesting that the improved insulin responsiveness in KLF14Tg mice may arise after a high‐fat diet challenge. However, this protection was transient and did not translate into sustained improvement in insulin sensitivity over time, as measured by the area under the curve (AUC) (Figure 2b).

Glucose tolerance tests were conducted at 21 weeks of age using glucose doses proportional to body weight, with blood glucose levels measured at baseline and 20, 40, 60, 90, and 120 min postinjection. Male KLF14Tg mice exhibited improved glucose clearance compared to WT males; whereas no differences were observed between genotypes in females (Figure 2c). Despite these transient differences, AUC analysis revealed no significant long‐term protection from glucose intolerance in either sex (Figure 2d).

Together, these results indicate that adipocyte‐specific Klf14 overexpression confers sex‐biased improvements in insulin and glucose sensitivity, with greater metabolic benefits observed in male mice during midlife.

### 3.3 KLF14Tg mice did not differ in whole‐body energy metabolism

To evaluate whole‐body energy metabolism, we conducted indirect calorimetry from 22 to 24 weeks of age in high‐fat diet–fed mice. Measurements of oxygen consumption, carbon dioxide production, and locomotor activity revealed no significant differences between KLF14Tg and WT mice (Figure S3). We further assessed RER, as well as inferred rates of carbohydrate and fat utilization. These parameters also remained unchanged across genotypes (Figure S4). These findings suggest that the protective metabolic effects of Klf14 overexpression are not mediated by alterations in basal energy expenditure or substrate preference.

### 3.4 KLF14Tg mice are not protected against diet‐induced serum lipid abundance

**FIGURE 3:**
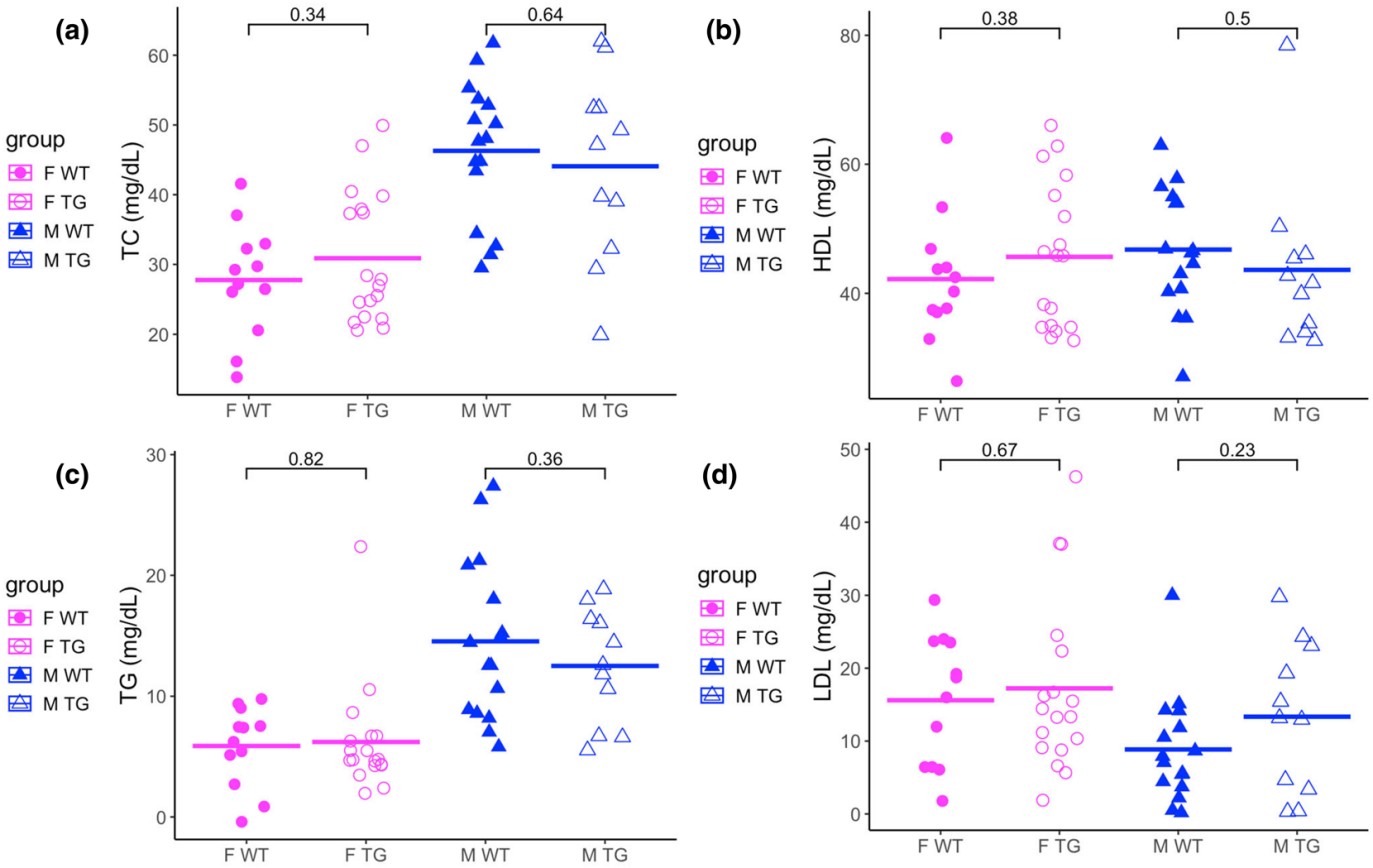
Transgenic mice do not differ from wild‐type littermates in serum lipid profiles. Serum was taken from F TG (*n* = 16), F WT (*n* = 12), M TG (*n* = 11), and M WT (*n* = 16) mice, and was assayed for (a) total cholesterol, (b) HDL levels, (c) TG, and (d) LDL levels. The mean is plotted with a horizontal bar, and differences were assessed with the Student's *t*‐test.

At the conclusion of the study, mice were sacrificed, and blood serum was collected to assess circulating lipid levels. Prior studies in both humans and mice have linked adipose‐specific *Klf14* deficiency with reduced high‐density lipoprotein (HDL) levels and elevated levels of low‐density lipoprotein (LDL), triglycerides (TG), and total cholesterol (TC). Based on these findings, we hypothesized that *Klf14* overexpression might enhance HDL abundance. However, serum lipid profiling revealed no significant differences in HDL levels between KLF14Tg and wild‐type (WT) mice (Figure 3a). Similarly, levels of TC, TG, and LDL were unchanged across genotypes (Figure 3b–d). These results suggest that adipocyte‐specific *Klf14* overexpression does not broadly alter systemic lipid profiles under high‐fat diet conditions.

### 3.5 KLF14Tg mice express greater levels of browning and lipid uptake genes

**FIGURE 4:**
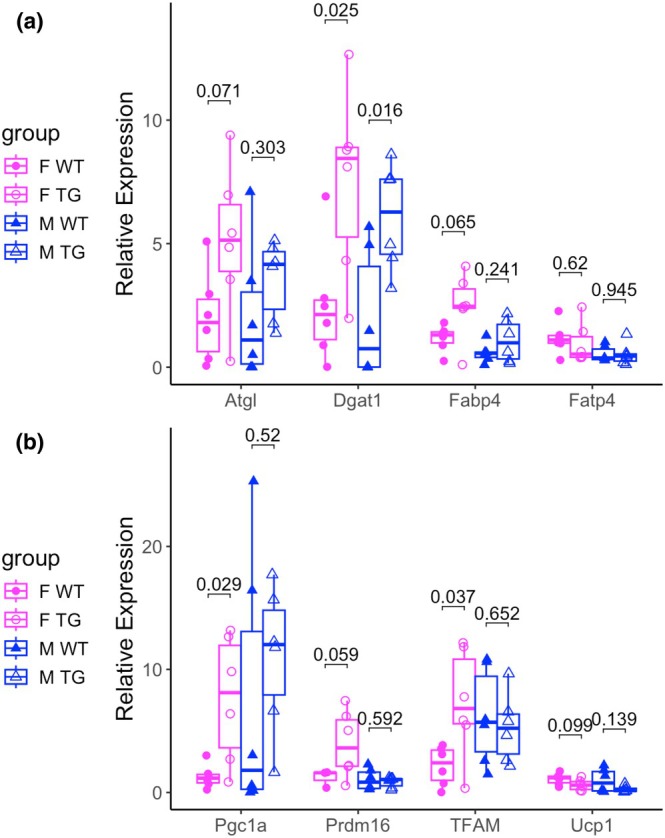
Female transgenic mice express more browning and lipid‐related genes in the subcutaneous adipose tissue (SAT). Relative expression of a panel of (a) lipolysis and lipid uptake genes and (b) browning genes in subcutaneous adipose tissue isolated from Klf14Tg mice (*n* = 5 in each group: F WT, F TG, M WT, M TG). The expression relative to the F WT expression is plotted with the median indicated by the horizontal bar. The interquartile range is plotted with the box, and data points for biological replicates are shown with dots. Relative expression was computed with the delta–delta Ct method; differences were assessed with the Student's *t*‐test.

Despite the absence of overt changes in serum lipid levels or whole‐body metabolic parameters, we hypothesized that transgene dosage in KLF14Tg mice might elicit transcriptional changes in key metabolic pathways. To investigate this, we performed RT–qPCR on RNA isolated from subcutaneous adipose tissue. In female KLF14Tg mice, we observed a trend toward increased expression of *Atgl* (encoding adipose triglyceride lipase) and *Fabp4* (fatty acid binding protein 4), with no change in *Fatp4* (long‐chain fatty acid transport protein 4) levels (Figure 4a). Across sexes, KLF14Tg mice exhibited elevated expression of *Dgat1* compared to wild‐type littermates (Figure 4a). Given the established roles of *Atgl*, *Fabp4*, and *Dgat1* in adipocyte lipolysis and lipid handling, these transcriptional changes suggest an upregulation of lipid storage and mobilization pathways in KLF14Tg adipose tissue, with a more pronounced effect in females.

We next assessed the expression of genes involved in adipose tissue browning. While Ucp1 levels were unchanged, the expression of upstream thermogenic regulators, including *Pgc1a* (Pparg coactivator 1 alpha), *Prdm16* (PR domain containing 16), and *TFAM* (mitochondrial transcription factor A), was significantly increased in Klf14Tg mice (Figure 4b). As Ucp1 is downstream of these factors and essential for non‐shivering thermogenesis, the absence of *Ucp1* induction aligns with our metabolic cage data, which showed no differences in energy expenditure. These findings suggest that although our level of *Klf14* overexpression primes the transcriptional landscape for adipose tissue browning, the absence of *Ucp1* activation may limit functional thermogenic output. Collectively, our data support a role for Klf14 in modulating adipocyte gene networks involved in lipid metabolism and browning, and indicate that higher expression levels may be required to elicit systemic metabolic phenotypes.

## 4 DISCUSSION

Obesity remains a significant risk factor for type 2 diabetes (T2D) and coronary artery disease (CAD). Weight loss interventions are required for the treatment of obesity. There are a few courses of action for these: bariatric surgery procedures, behavioral modification of diet and exercise, GLP‐1 receptor agonists (GLP1‐RAs), and SGLT2 inhibitors (SGLT2‐is) (Elmaleh‐Sachs et al., 2023). The advent of these therapeutic interventions has offered powerful tools to clinicians and patients, but approximately half of the users of SGLT2‐is and GLP1‐RAs discontinued therapy within 5 years (Malik et al., 2023). SGLT2 inhibitors significantly increase insulin sensitivity, but their effect on weight loss is much more modest than GLP‐1 receptor agonists. Behavioral interventions can have durable effects, but dropout is significant. Therefore, it is critical to continue developing more treatments for obesity that can improve healthcare outcomes of patients unable to adhere to any pharmacological interventions. Given the sex differences in adipose tissue distribution and function, we included well‐powered, sex‐balanced cohorts to assess whether mature adipocyte KLF14 overexpression produces sex‐biased metabolic effects. We find protection from weight gain and adiposity in female KLF14Tg mice but not in male mice (Figure 1). Despite this, we find that male KLF14Tg mice are protected from acute insulin and glucose resistance, but female KLF14Tg mice are not (Figure 2). KLF14Tg mice do not appear different in serum lipid levels (Figure 3) or whole‐body metabolism (Figures S3 and S4). Organ weights do not differ between wild‐type and KLF14Tg mice (Figure S1). Despite the lack of serum lipid profile changes and energy metabolism changes, we found that *Dgat1*, *Tfam*, *Pgc1a*, and *Prdm16* were more abundantly expressed in female KLF14Tg mice than in WT littermates (Figure 4). Overall, these results indicate modest protection in KLF14Tg mice, with sex‐specific phenotypes in weight gain and insulin resistance.

The modest protection may be explained by dosage effects. Our previous work characterized the metabolic effects of adipocyte‐specific Klf14 knockout (KLF14‐KO) in mice (Yang et al., 2022). We observed no significant metabolic differences between male WT and male KLF14‐KO mice. In contrast, we observed several metabolically deleterious effects in female KLF14‐KO mice compared to WT littermates. Female KLF14‐KO mice exhibited greater adiposity, insulin resistance, elevated serum triglyceride levels, and fat storage shifts to the visceral depots from the subcutaneous fat depots. We observed increased expression of similar lipid packaging marker genes that were decreased in KLF14‐KO mice, supporting the role of KLF14 in maintaining the expression of genes promoting lipid metabolism. Mao et al. used an adenoviral overexpression system with a CMV promoter to induce adipose tissue overexpression of KLF14 at 40 times over wild type (Mao et al., 2023). They observed adipose tissue browning in KLF14 overexpression mice. This supraphysiological dose of KLF14 expression was targeted to adipose tissue, but not to any particular cell type, so it is difficult to conclude that only the increased expression of KLF14 in mature adipocytes contributed to adipose tissue browning. Compared to the adipocyte‐specific 1.7‐fold overexpression in the present study, closer fold changes in expression resulting from genetic variation (Mohammadi et al., 2017), these perturbations were much stronger and broader. This discrepancy may explain the modest phenotypes observed in our study in insulin resistance, serum lipid levels, and indirect calorimetry. Of note, AAV‐mediated gene expression is typically episomal (Brommel et al., 2020), meaning the expression might be transient and non‐nuclear. There is a significant need to validate, such as with chromatin‐immunoprecipitation (ChIP) and RNA sequencing, that AAV delivery of transcription factors induces typical transcriptional regulation. Additionally, Mao et al. included only male mice in their study, but still found metabolic protection. They observed that KLF14‐AAV mice gained less weight, had greater expression of browning genes, had decreased markers of hepatic steatosis, had improved glycemic tolerances, and had smaller adipocytes (Mao et al., 2023). We find elevated expression of similar markers of lipid packaging and respiration in female Klf14Tg mice, but not in male Klf14Tg mice. Notably, we do not find differences in *Ucp1* expression, which implies classical browning is not involved in phenotypic differences in Klf14Tg mice. Human and mouse adipose tissue browning differ in many respects, including the distribution of brown fat depots, beiging capability of white fat, and thermogenic capacity (Herz & Kiefer, 2019). While greater levels of human subcutaneous adipose tissue KLF14 mRNA expression are associated with greater UCP1 expression (Mao et al., 2023), there is a need for mechanistic studies to follow up on this relationship in humans.

Investigation of modulating KLF14 in other tissues and cell types similarly reveals potential effects of KLF14 in male mice. Induction or inhibition of KLF14 expression with perhexiline malleate or EPZ‐6438, respectively, showed directionally consistent results. KLF14 expression was critical to maintaining hepatic gluconeogenesis and reducing hepatic fibrosis in male mice (Du et al., 2021; Guo et al., 2015; Wang et al., 2017). Wei et al. found that aortic tissue isolated from male *ApoE* knockout mice expresses more *KLF14* than WT littermates on HFD and that shRNA‐mediated knockdown of KLF14 in the aortic tissue led to greater atherosclerotic lesion development and reduced inflammatory cytokine secretion in male mice (Wei et al., 2017). These studies must be performed in female mice to understand if the sex‐dependent effect of KLF14 expression is adipose‐specific or persistent across tissues and cell types. Still, most investigations into KLF14 in adipose tissue point to greater effectiveness in female mice than in male mice (Argmann et al., 2017; Yang et al., 2022; Yang & Civelek, 2020). In agreement with rodent studies, human genetics findings also point to more beneficial effects of increasing KLF14 expression in women than in men (Small et al., 2018).

Further investigation of KLF14‐based therapeutics is a promising endeavor. KLF14, a transcription factor, likely affects weight gain by regulating primary transcriptional targets related to metabolism. Thus, the identification of these primary targets will reveal the specific mechanisms through which KLF14 has pleiotropic effects across metabolic phenotypes. Because ChIP of KLF14 is difficult in adipocytes, using novel pulldown and sequencing methods will be required to identify targets (Hou et al., 2023). Additionally, these might reveal the specific mechanism through which sex‐biased effects of KLF14 modulation are observed. Finally, the drug perhexilline malleate, a CPT1 inhibitor, has been observed to increase KLF14 expression (Guo et al., 2015). Perhexilline has toxic systemic effects, and developing adipocyte‐specific drug delivery methods can reduce the off‐target effects of perhexilline while improving the ability to administer higher doses of perhexilline for greater KLF14 expression. Drug delivery to adipose tissue is challenging, but advances have been made in recent years using novel drug‐loaded nanoparticle delivery systems (Kupor et al., 2024; Safari et al., 2020). This investigation contributes to the growing body of evidence that KLF14 and its targets influence adipose metabolism in sex‐biased ways, highlighting the importance of their study for the development of metabolic therapeutics.

## FUNDING INFORMATION

This work was supported by the National Heart, Lung, and Blood Institute (2T32HL007284 to Y.T.A. and 5R25HL088724 to D.G.D.), the National Science Foundation (2447802 to F.K.), the National Institute of Diabetes and Digestive and Kidney Diseases (R01 DK118287 to M.C.), the American Diabetes Association (1‐19‐IBS‐105 to M.C.), and the American Heart Association (24EIA1258067 to M.C.).

## ETHICS STATEMENT

The study protocols involving mice were reviewed and approved by the University of Virginia Institutional Animal Care and Use Committee (IACUC), under protocol number 4103. Mice were housed and treated in accordance with the NIH Guide for the Care and Use of Laboratory Animals, maintaining standards for welfare and minimizing distress. Sacrifices were performed using methods approved for rodents, consistent with recommendations to ensure humane endpoints and appropriate euthanasia protocols.

## Supporting information


Figure S1.

Figure S2.

Figure S3.

Figure S4.

Table S1.

Table S2.

Table S3.

## Data Availability

The raw data and the statistical analysis pipelines are available on GitHub (https://github.com/aberrations/Klf14Tg).
