# Supplementary material for: Adipocyte‐specific Krüppel‐like factor 14 overexpression confers sex‐biased protection from weight gain on a high‐fat diet
Source: Physiol Rep. 2025 Aug 11;13(15):e70513. doi: 10.14814/phy2.70513 (PMC12339416; doi:10.14814/phy2.70513)
Supplement: Supplementary file 1 — Figure S1. [file PHY2-13-e70513-s007.docx]

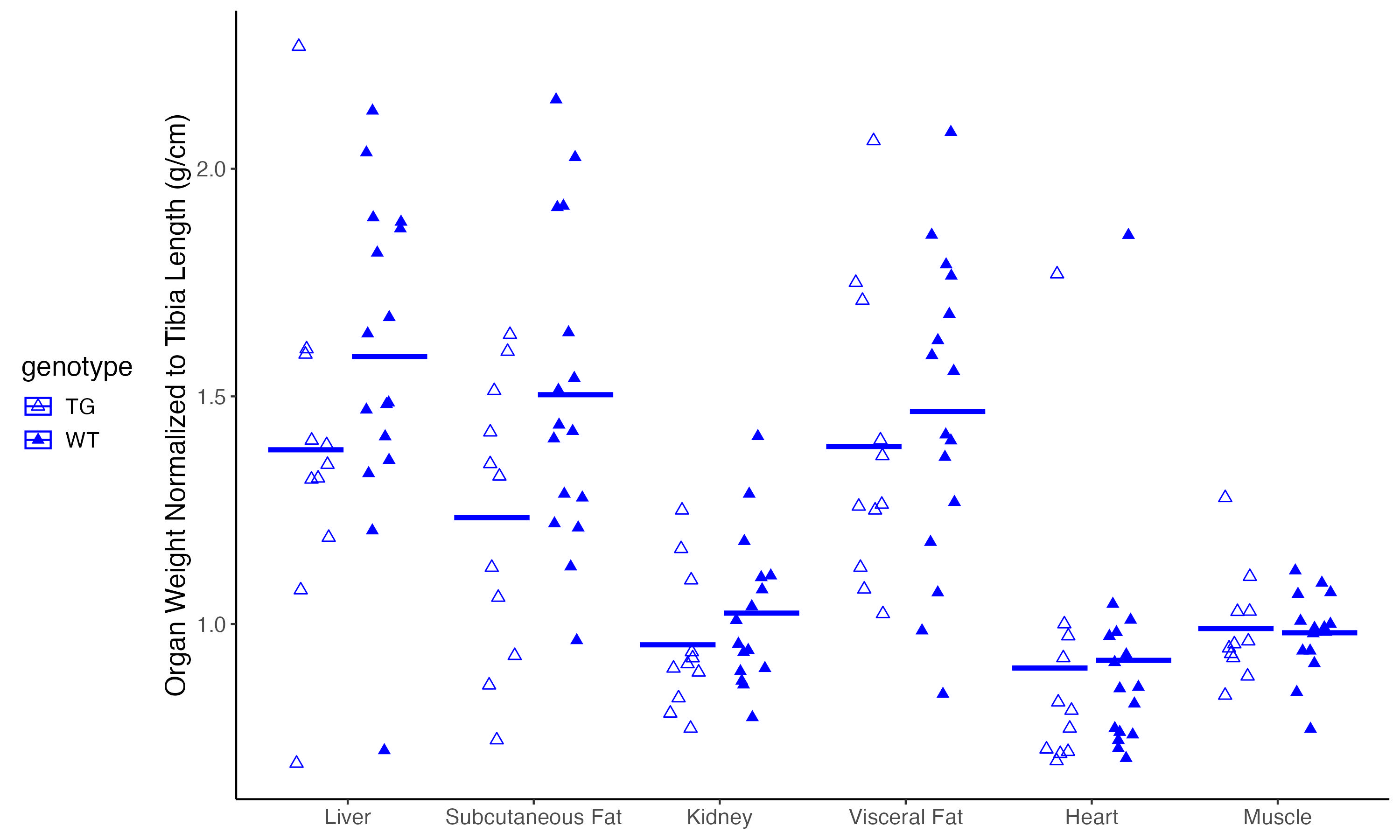

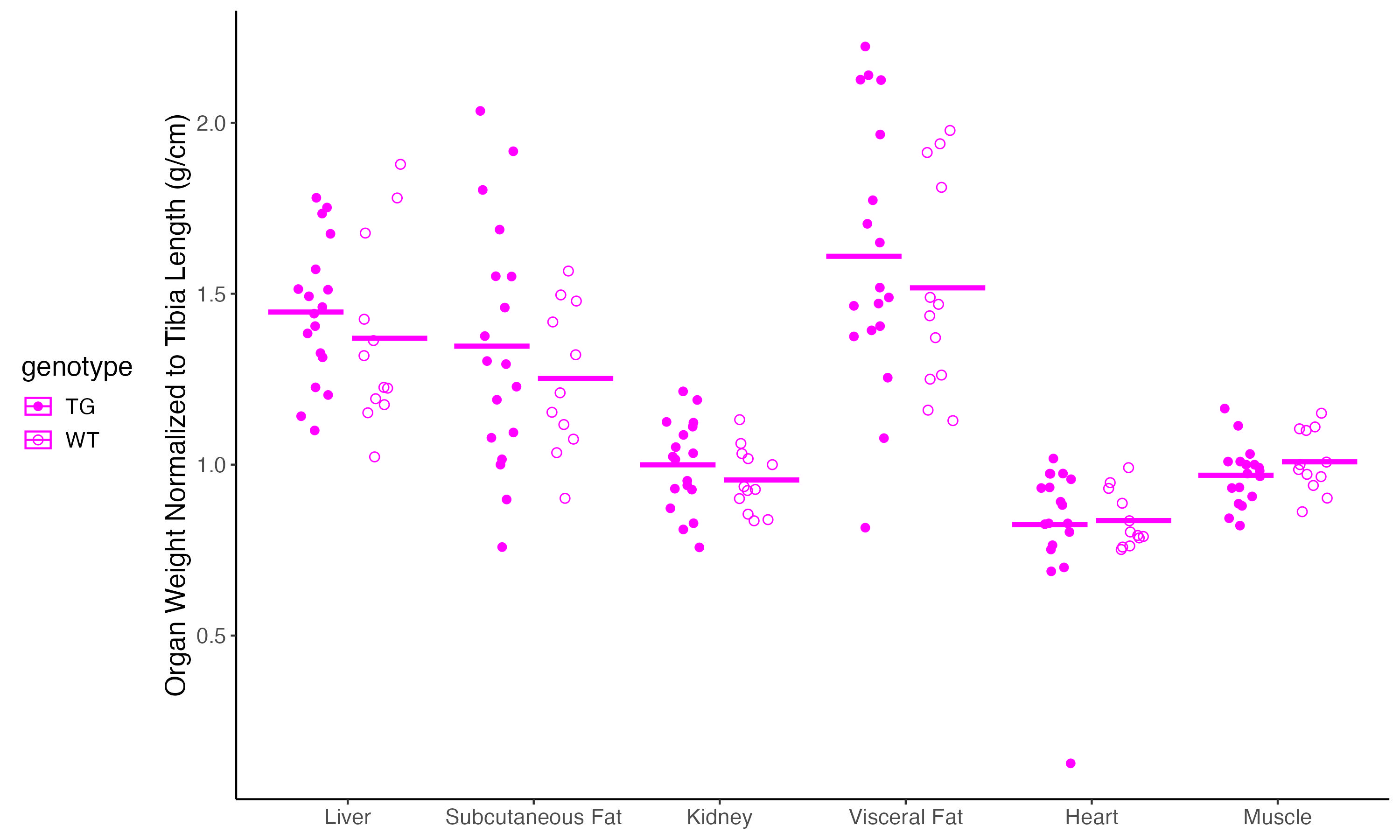


A.

B.

**Supplementary Figure S1**. KLF14Tg mice have kidneys, hearts, liver, and skeletal muscle of similar weight to WT mice. At week 48, we weighed organs of **(A)** M TG (*n* = 11) and M WT (*n* = 16) mice, and **(B)** F TG (*n* = 16) and F WT (*n* = 12) mice. The mean is plotted with the horizontal bar along with individual data points. Differences were assessed with the Student’s t-test.
