# Supplementary material for: Adipocyte‐specific Krüppel‐like factor 14 overexpression confers sex‐biased protection from weight gain on a high‐fat diet
Source: Physiol Rep. 2025 Aug 11;13(15):e70513. doi: 10.14814/phy2.70513 (PMC12339416; doi:10.14814/phy2.70513)
Supplement: Supplementary file 2 — Figure S2. [file PHY2-13-e70513-s001.docx]


**Supplemental Figure S2.** Klf14Tg mice do not differ from wild-type littermates in insulin tolerance after 5 weeks of high-fat diet. **(A)** The mean of blood glucose levels, normalized to the initial time point, is plotted, along with the standard error of the mean. **(B)** The area under the curve (AUC) for the time-course blood glucose levels in the insulin tolerance test. The glucose tolerance test was performed on F TG (*n* = 16), F WT (*n* = 12), M TG (*n* = 11), and M WT (*n* = 16) mice. Differences in time-course glucose levels were assessed with the repeated measures ANOVA with terms for sex, genotype, time, and interactions between these. This was followed by Tukey’s test comparing wild-type and transgenic mice within sex. Differences in the AUC glucose levels were assessed with the repeated measures ANOVA, followed by Tukey’s test comparing wild-type and transgenic mice within sex.
