# Supplementary material for: Adipocyte‐specific Krüppel‐like factor 14 overexpression confers sex‐biased protection from weight gain on a high‐fat diet
Source: Physiol Rep. 2025 Aug 11;13(15):e70513. doi: 10.14814/phy2.70513 (PMC12339416; doi:10.14814/phy2.70513)
Supplement: Supplementary file 3 — Figure S3. [file PHY2-13-e70513-s006.docx]


**Supplemental Figure S3.** Klf14Tg mice do not differ in motor activity, oxygen consumption, or carbon dioxide compared to wild-type littermates. F TG (*n* = 16), F WT (*n* = 12), M TG (*n* = 11), and M WT (*n* = 16) mice were placed in metabolic cages, and **(A)** diurnal locomotor activity, **(B)** diurnal O_2_ consumption, **(C)** diurnal CO_2_ production, **(D)** nocturnal locomotor activity, **(E)** nocturnal O_2_ consumption, and **(F)** nocturnal CO_2_ production were assayed. The mean is plotted with a horizontal bar, and differences were assessed using a two-way ANOVA with terms for sex, genotype, and the interaction between sex and genotype.
