# Supplementary material for: Adipocyte‐specific Krüppel‐like factor 14 overexpression confers sex‐biased protection from weight gain on a high‐fat diet
Source: Physiol Rep. 2025 Aug 11;13(15):e70513. doi: 10.14814/phy2.70513 (PMC12339416; doi:10.14814/phy2.70513)
Supplement: Supplementary file 4 — Figure S4. [file PHY2-13-e70513-s004.docx]

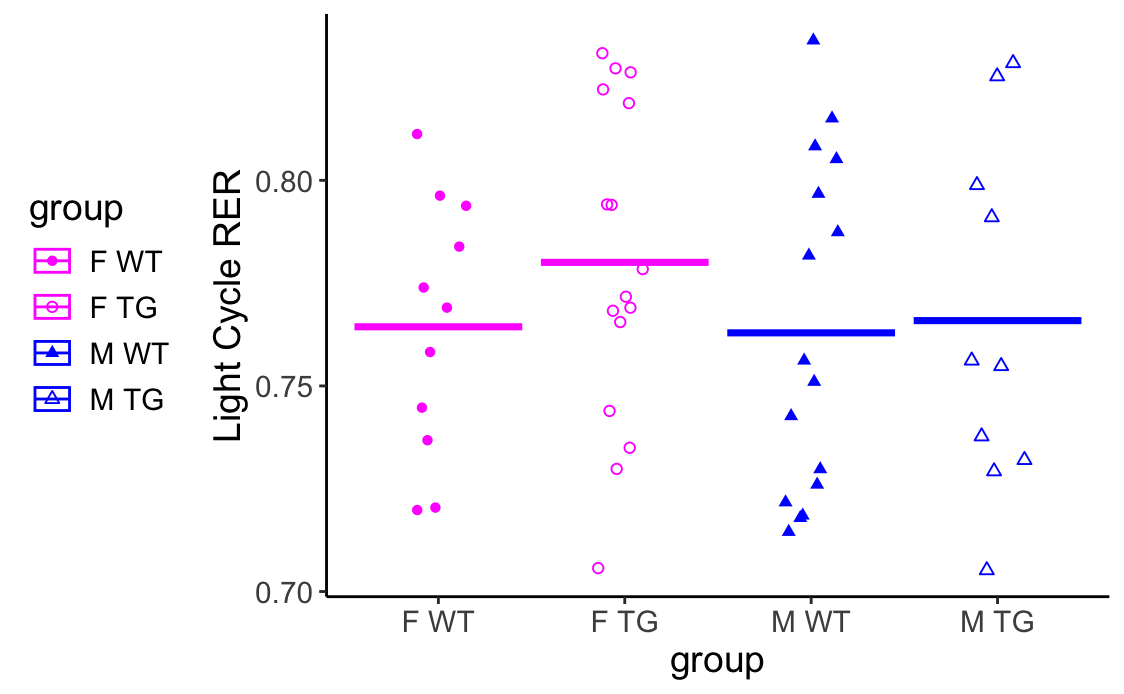

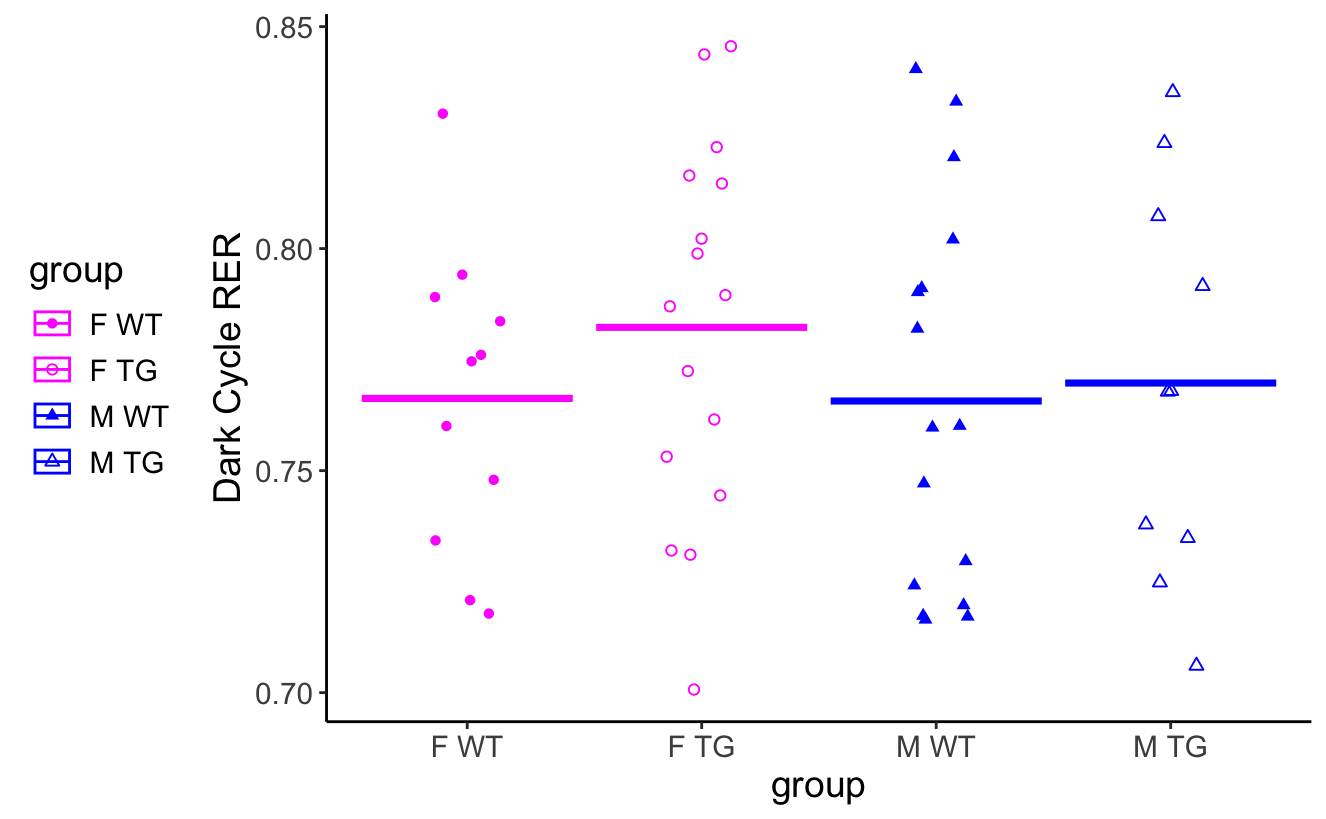

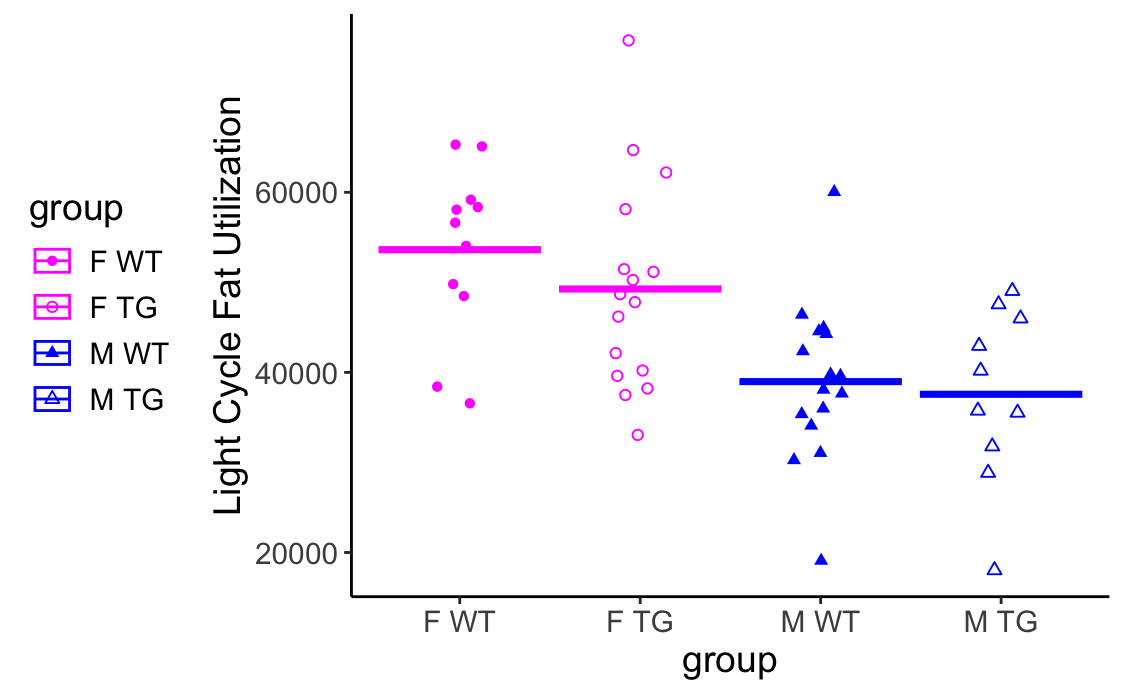

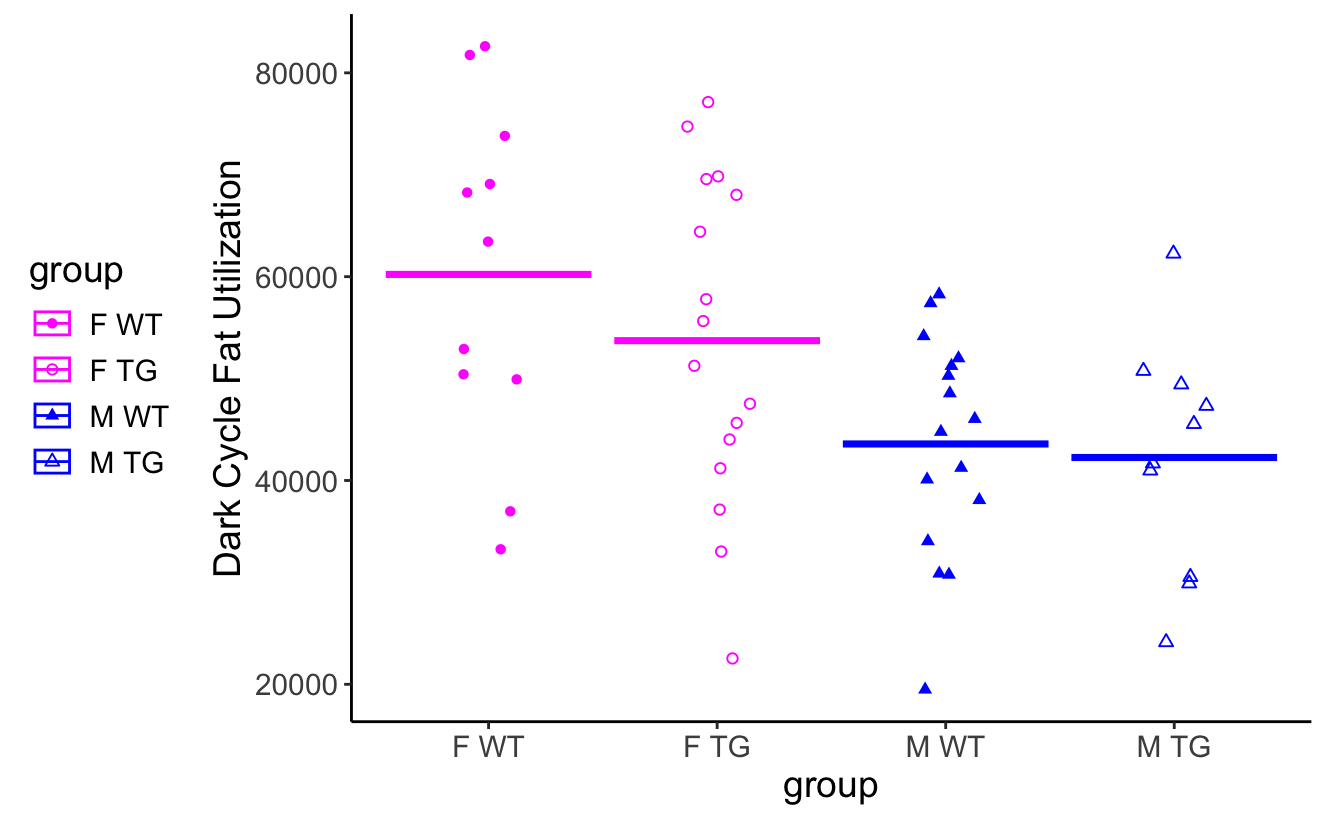

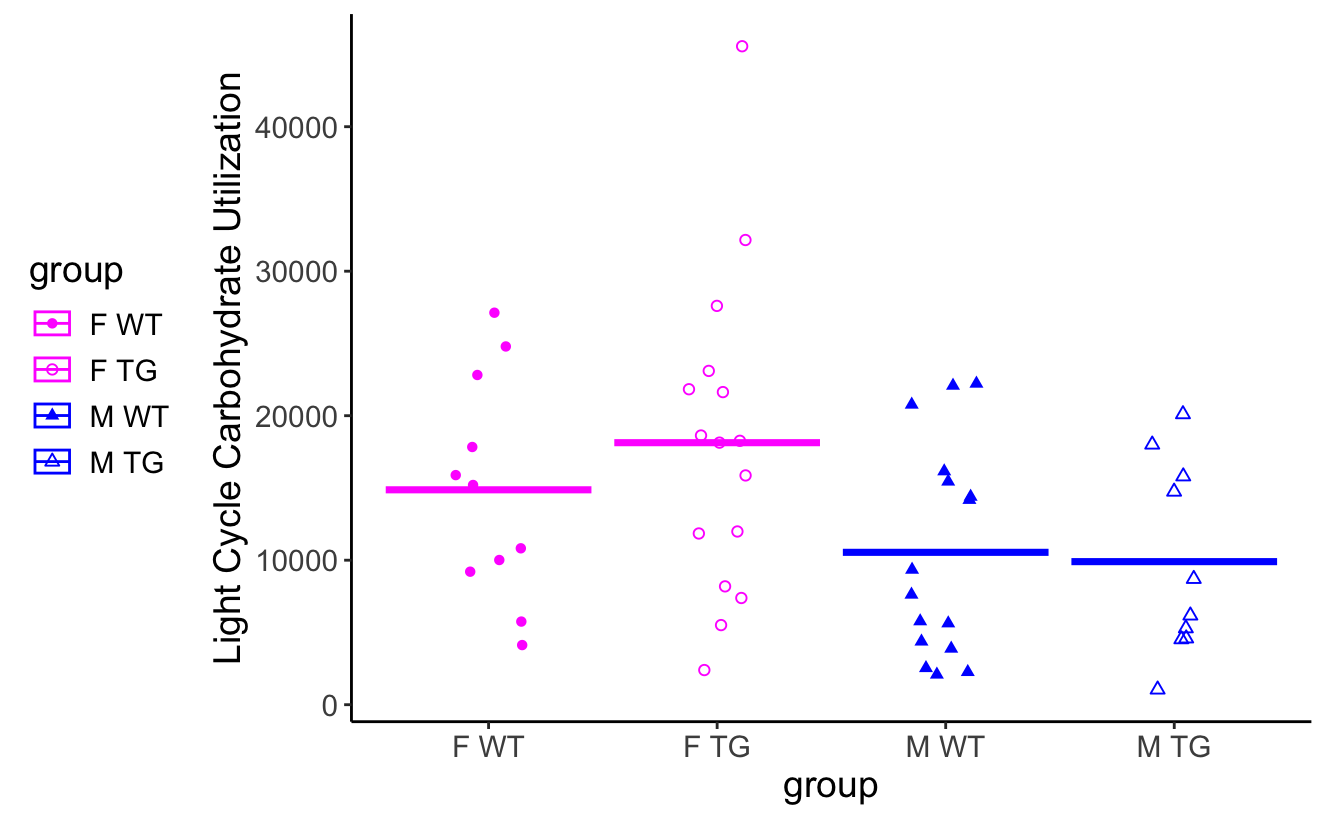

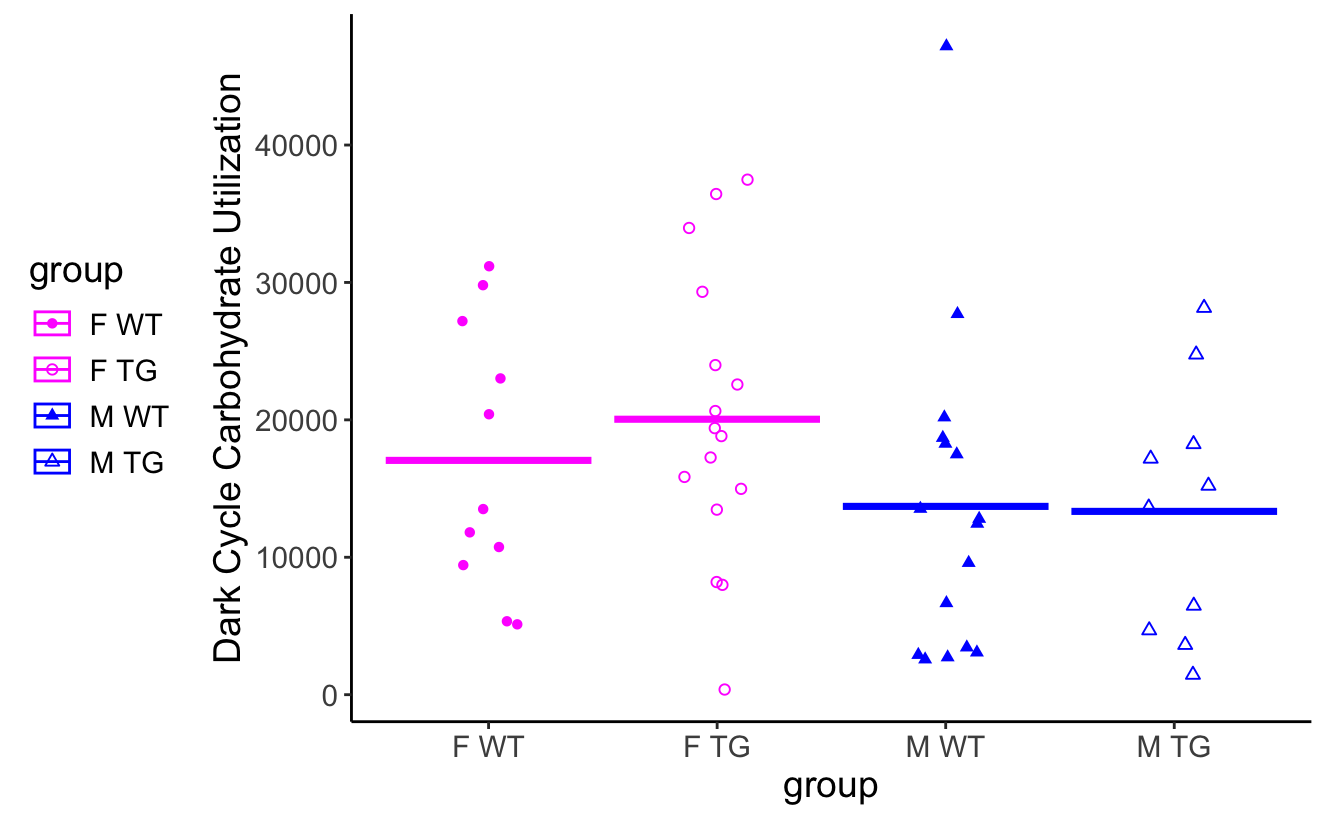


A.

B.

C.

D.

E.

F.

**Supplemental Figure S4.** Klf14Tg mice do not differ in energy metabolism compared to wild-type littermates. F TG (*n* = 16), F WT (*n* = 12), M TG (*n* = 11), and M WT (*n* = 16) mice were placed in metabolic cages, and (A) diurnal RER, (B) diurnal fat utilization, (C) diurnal carbohydrate utilization, (D) nocturnal RER, (E) nocturnal fat utilization, and (F) nocturnal carbohydrate utilization were assayed. The mean is plotted with a horizontal bar, and differences were assessed using a two-way ANOVA with terms for sex, genotype, and the interaction between sex and genotype.
