# Supplementary material for: Adipocyte‐specific Krüppel‐like factor 14 overexpression confers sex‐biased protection from weight gain on a high‐fat diet
Source: Physiol Rep. 2025 Aug 11;13(15):e70513. doi: 10.14814/phy2.70513 (PMC12339416; doi:10.14814/phy2.70513)
Supplement: Supplementary file 5 — Table S1. [file PHY2-13-e70513-s003.docx]

**Supplementary Table S1: Mouse genotyping primer list.**

| **Primer** | **Sequence** |
| --- | --- |
| Adpq_Klf14Tg_390F | GGCCTACTACAAGTCGTCGC |
| Adpq_Klf14Tg_390R | CCGGGCTGCAGGAATTCGAT |
| Adpq_Klf14Tg_582F | CAGTGGATCTGACGACACCAA |
| Adpq_Klf14Tg_582R | GGCAGCGAAGTAGTCCAGG |
