# Supplementary material for: Adipocyte‐specific Krüppel‐like factor 14 overexpression confers sex‐biased protection from weight gain on a high‐fat diet
Source: Physiol Rep. 2025 Aug 11;13(15):e70513. doi: 10.14814/phy2.70513 (PMC12339416; doi:10.14814/phy2.70513)
Supplement: Supplementary file 6 — Table S2. [file PHY2-13-e70513-s002.docx]

**Supplementary Table S2: Mouse cohort sex and genotype data.**

| **Mouse ID** | **Sex** | **Genotype** |
| --- | --- | --- |
| 232LC | F | WT |
| 232RC | F | WT |
| 232BC | F | TG |
| 232LP | F | TG |
| 233LC | F | TG |
| 233RC | F | TG |
| 233BC | F | WT |
| 234LC | M | TG |
| 234RC | M | WT |
| 234BC | M | TG |
| 234LP | M | WT |
| 234RP | M | WT |
| 236LC | F | TG |
| 236RC | F | WT |
| 236BC | F | TG |
| 236LCRP | F | WT |
| 236RP | F | TG |
| 237LC | M | TG |
| 237RC | M | WT |
| 237BC | M | TG |
| 237LP | M | TG |
| 237RP | M | TG |
| 238LC | F | TG |
| 238RC | F | WT |
| 238BC | F | TG |
| 239RC | M | WT |
| 239BC | M | WT |
| 239LP | M | TG |
| 239RP | M | WT |
| 240LC | M | WT |
| 240RC | M | TG |
| 240LP | M | WT |
| 241LC | M | WT |
| 241RC | M | WT |
| 241BC | M | TG |
| 242LC | M | TG |
| 242RC | M | WT |
| 242BC | M | WT |
| 242RP | M | WT |
| 243LC | F | WT |
| 243RC | F | WT |
| 243BC | F | TG |
| 243LP | F | TG |
| 243RP | F | WT |
| 244LC | M | TG |
| 244RC | M | WT |
| 244BC | M | WT |
| 245LC | F | TG |
| 245RC | F | TG |
| 245BC | F | TG |
| 245LP | F | WT |
| 245RP | F | WT |
| 247RC | F | TG |
| 247LC | F | WT |
| 247BC | F | TG |
