# Supplementary material for: Adipocyte‐specific Krüppel‐like factor 14 overexpression confers sex‐biased protection from weight gain on a high‐fat diet
Source: Physiol Rep. 2025 Aug 11;13(15):e70513. doi: 10.14814/phy2.70513 (PMC12339416; doi:10.14814/phy2.70513)
Supplement: Supplementary file 7 — Table S3. [file PHY2-13-e70513-s005.docx]

**Supplementary Table S3: RT-qPCR primer list.**

| **Gene** | **Forward primer** | **Reverse primer** | **Source** |
| --- | --- | --- | --- |
| Gapdh | CTCCCACTCTTCCACCTTCG | GCCTCTCTTGCTCAGTGTCC | ^1^ |
| Atgl | TGTGGCCTCATTCCTCCTAC | TCGTGGATGTTGGTGGAGCT | ^2^ |
| Dgat1 | ACCGCGAGTTCTACAGAGATTGGT | ACAGCTGCATTGCCATAGTTCCCT | ^3^ |
| Fatp4 | ACGATGTTTCCTGCTGAGTGGTA | CTCTCCGACCTGCCACAGA | ^4^ |
| Fabp4 | ATGTGCGACCAGTTTGTG | TTTGCCATCCCACTTCTG | ^5^ |
| Ucp1 | AGGCTTCCAGTACCATTAGGT | CTGAGTGAGGCAAAGCTGATTT | ^6^ |
| Prdm16 | CCAAGGCAAGGGCGAAGAA | AGTCTGGTGGGATTGGAATGT | ^6^ |
| PGC1α | GGTTGAAAAAGCTTGACTGGCG | ACCAACCAGAGCAGCACACT | ^6^ |
| TFAM | TCCCCTCGTCTATCAGTCTTGTC | AATTTGGGTAGCTGTTCTGTGG | ^6^ |

**References**

1. Ruiz-Villalba, A. *et al.* Reference genes for gene expression studies in the mouse heart. *Sci Rep* **7**, 24 (2017).

2. Reid, B. N. *et al.* Hepatic Overexpression of Hormone-sensitive Lipase and Adipose Triglyceride Lipase Promotes Fatty Acid Oxidation, Stimulates Direct Release of Free Fatty Acids, and Ameliorates Steatosis. *J Biol Chem* **283**, 13087–13099 (2008).

3. Lee, B., Fast, A. M., Zhu, J., Cheng, J.-X. & Buhman, K. K. Intestine-specific expression of acyl CoA:diacylglycerol acyltransferase 1 reverses resistance to diet-induced hepatic steatosis and obesity in Dgat1−/− mice. *J Lipid Res* **51**, 1770–1780 (2010).

4. Mishima, T., Miner, J. H., Morizane, M., Stahl, A. & Sadovsky, Y. The Expression and Function of Fatty Acid Transport Protein-2 and -4 in the Murine Placenta. *PLoS One* **6**, e25865 (2011).

5. Gan, L., Liu, Z., Cao, W., Zhang, Z. & Sun, C. FABP4 reversed the regulation of leptin on mitochondrial fatty acid oxidation in mice adipocytes. *Sci Rep* **5**, 13588 (2015).

6. Mao, X. *et al.* Kruppel-like factor 14 ameliorated obesity and related metabolic disorders by promoting adipose tissue browning. *American Journal of Physiology-Endocrinology and Metabolism* **325**, E744–E754 (2023).
